# Coagulation-related genes COL3A1 and MMP1 influence the development of osteoarthritis and the surrounding immunological environment

**DOI:** 10.3389/fimmu.2026.1759281

**Published:** 2026-01-28

**Authors:** Liangkun Huang, Xuezhong Wang, Zijie Pei, Ze Zhang, Fengpo Sun, Liangyuan Wen

**Affiliations:** 1 Beijing Hospital, National Center of Gerontology, Institute of Geriatric Medicine, Chinese Academy of Medical Science & Peking Union Medical College, Beijing, China; 2 Department of Orthopedics, Tongji Hospital, Tongji Medical College, Huazhong University of Science and Technology, Wuhan, China

**Keywords:** coagulation system, immune microenvironment, machine learning, osteoarthritis, protein interaction analysis

## Abstract

**Background:**

Coagulation is an important physiological process for the body to cope with vascular injury, involving platelet activation, coagulation factor cascade reaction and fibrin formation. The role of the coagulation system in inflammatory and degenerative diseases has received increasing attention in recent years. However, its impact for osteoarthritis remains to be well investigated.

**Methods:**

The GEO database provided us with microarray data that included osteoarthritis and normal samples. The Genecards database provided coagulation-related genes. Protein interaction network analysis, machine learning, and screening for differentially expressed genes were used to identify coagulation-related core genes relevant to osteoarthritis. Coagulation-related osteoarthritis subtypes were identified by clustering analysis. Enrichment analysis and immune infiltration analysis revealed the potential mechanism of coagulation-related genes promoting osteoarthritis progression. The screened core genes were further validated by chondrocyte experiments.

**Result:**

We successfully screened the coagulation-related genes COL3A1 and MMP1 as core genes for osteoarthritis diagnosis. Both nomogram and diagnostic model constructed based on them have excellent diagnostic value, while OA samples can be classified into different subtypes. Immune infiltration study confirmed enrichment analysis’s finding that COL3A1 could affect the course of osteoarthritis by controlling immunological pathways. Basic research confirms that overexpression of COL3A1 inhibits proliferation and viability of chondrocytes and promotes senescence and damage. We confirmed that COL3A1 is an intervention target for osteoarthritis.

**Conclusion:**

Our study identifies osteoarthritis subtypes associated with coagulation and reveals the regulatory role of COL3A1 on chondrocytes in inflammatory environment. It offers fresh perspectives on osteoarthritis management.

## Introduction

Osteoarthritis (OA) has become a prevalent progressive arthritis that significantly lowers patients’ quality of life. It is identified by osteophytes, synovial inflammation, and articular cartilage breakdown (1). Globally, OA is much more common as people age, particularly in the elderly, where the prevalence is up to more than 10% (2). In China, the number of OA patients has exceeded 130 million, and this number continues to grow with the aging of the population (3). Numerous variables, including biomechanics, inflammatory response, metabolic abnormalities and immune regulation, are involved in the complicated etiology of OA (4). Currently, therapeutic strategies for OA include pharmacologic therapy (e.g., NSAIDs, glucocorticosteroids), physical therapy (e.g., exercise therapy, hot packs), and surgical treatment (e.g., joint replacement) (5, 6). However, most of the existing treatments focus on symptomatic relief and fail to fundamentally reverse cartilage damage or stop disease progression (7). Therefore, exploring new therapeutic targets and mechanisms has become an important direction in current OA research. Coagulation is an important physiological process for the body to cope with vascular injury, involving platelet activation, coagulation factor cascade reaction and fibrin formation (8). Recently, the coagulation system’s function in non-vascular diseases has received increasing attention, especially for its potential function in inflammatory and degenerative diseases (9–11). Studies have shown that thrombin, a key enzyme in the coagulation process, is not only involved in hemostasis, but also regulates the inflammatory response, cell proliferation and tissue repair through the activation of protease-activated receptors (PARs) (12–14). Clinical epidemiology further supports this link, as use of vitamin K antagonists (which impair the function of coagulation-related Gla proteins) is associated with an increased risk of osteoarthritis incidence and progression (15). At the molecular level, proteomic analyses have identified a diagnostic signature for knee OA, with the involved proteins significantly enriched in the complement and coagulation pathways, highlighting the cross-talk between coagulation and inflammation in OA pathogenesis (16). Additionally, it has been shown that fibrin plaques directly compromise cartilage integrity in arthritis through a triad of catabolism, adhesion and calcification (17). However, existing studies on the association involving coagulation-associated genes as well as osteoarthritis development have not been fully elucidated, thus the subject needs to be further investigated. In this study, we adopted a broad perspective on “coagulation-related” pathology, focusing not only on canonical coagulation cascade components but also on genes that interact with the coagulation system or participate in the coagulation-inflammation-fibrosis cross-talk, a key pathological network in degenerative joint diseases (17, 18). We hypothesized that key genes operating within this network could serve as robust biomarkers and stratify OA into subtypes with distinct pathological features. Notably, our core genes, COL3A1 and MMP1, exemplify this concept: COL3A1, as a major vascular matrix protein, provides the scaffold for platelet adhesion and thrombus formation (19, 20), while MMP1 can directly activate protease-activated receptor 1 (PAR1), a key thrombin receptor, thereby bridging extracellular matrix degradation and coagulation-associated signaling (21). Therefore, the importance of coagulation-related genes in OA prevention, diagnosis, and therapy was the main focus of our investigation, and we have explored targets for intervention in osteoarthritis. We discovered the significance of coagulation-associated genes COL3A1 and MMP1 on OA typing and immunity prediction, and experimentally verified the effect of COL3A1 on the properties of chondrocytes and cartilage tissues, our results suggest that COL3A1 is a crucial target for OA diagnosis and treatment.

## Materials and methods

### Collecting datasets

Gene expression profiles were obtained from the Gene Expression Omnibus (GEO) database. Five publicly available microarray datasets were included: GSE55235, GSE55457, and GSE82107 (derived from synovial tissue), and GSE51588 and GSE169077 (derived from articular cartilage). In total, these datasets comprised 76 osteoarthritis (OA) samples and 42 healthy control samples (As shown in **Table 1**). A list of 1,584 coagulation-related genes (CRGs) was retrieved from the GeneCards database (https://www.genecards.org/), including genes with a “Relevance score” > 1 that encode proteins involved in coagulation pathways. This threshold was applied to select genes with at least one or more lines of established evidence supporting their involvement in coagulation pathways, thereby constructing a biologically relevant and high-confidence candidate gene set for subsequent analysis (22, 23). To construct analysis cohorts that are representative of the joint microenvironment and to control for potential bias arising from tissue heterogeneity, we designed both the training set and the independent test set to contain a mixture of cartilage and synovial tissue samples. For cohort construction, expression matrices from GSE51588 (cartilage), GSE55457 (synovium), and GSE55235 (synovium) were merged and integrated using the removeBatchEffect function from the limma R package to form the training set. Similarly, GSE169077 (cartilage) and GSE82107 (synovium) were merged and batch-corrected using the same procedure to form the independent test set. The successful removal of batch effects was confirmed by comparative box plots of normalized expression values across the integrated datasets.

**Table 1:** Baseline characteristics of the OA dataset.

Group	Data set	Microarray platform	OA sample size	Normal sample size	Species	Tissue sources
*Training sets*	*GSE55457*	*GPL96*	*10*	*10*	*Homo sapiens*	*Synovium*
	*GSE55235*	*GPL96*	*10*	*10*	*Homo sapiens*	*Synovium*
	*GSE51588*	*GPL13497*	*40*	*10*	*Homo sapiens*	*Cartilage*
*Test sets*	*GSE169077*	*GPL96*	*6*	*5*	*Homo sapiens*	*Cartilage*
	*GSE82107*	*GPL570*	*10*	*7*	*Homo sapiens*	*Synovium*

### Acquisition of coagulation-related differentially expressed genes

Using the R statistical tool “limma,” we carried out differential expression analysis comparing healthy and normal control samples with OA samples in the training set to identify differentially expressed genes (DEGs). Volcano plot of DEGs was drawn. The CRGs and DEGs were taken to intersect to obtain coagulation-related differentially expressed genes (DECRGs). Protein interaction analysis and visualization of DECRGs were performed using string database and cytoscape software.

### Access to diagnostic core genes for OA

We employed three distinct machine learning algorithms—Generalized Linear Model (GLM), Support Vector Machine (SVM), and Random Forest (RF)—to screen differentially expressed coagulation-related genes (DECRGs). To identify the most robust signals, the top 50 genes ranked by importance from each algorithm were selected for intersection. This cutoff was chosen because a clear inflection point was observed around the top 50 genes in the importance ranking curves across all methods, beyond which scores plateaued. This approach captures the most discriminatory features while focusing the consensus analysis on high-potential candidates (24). The protein-protein interaction (PPI) network centrality measures (Betweenness, MNC, Eccentricity) were calculated, and genes ranking in the top 50% for each measure were selected to capture topologically important nodes. The final set of core diagnostic genes was determined by taking the intersection of the gene sets derived from the machine learning consensus and the PPI network centrality analysis. This stringent, multi-perspective approach ensured the identification of robust biomarkers.

### Construction of OA diagnostic model and nomogram

Violin plots for differential expression were generated using ggstatsplot. The area under the receiver operating characteristic curve (AUC) was calculated for each candidate gene using the ROCR package. Genes with an AUC greater than 0.7 were selected to build the diagnostic model. This widely adopted threshold ensures that only features with at least “acceptable” discriminatory power (AUC 0.7-0.8) are included, enhancing the model’s foundational validity (25–27). A multivariate logistic regression model was then built using the glmnet package with these selected genes. The model’s performance was evaluated by computing its AUC in both training and test sets. Finally, a nomogram was developed and calibrated using the rmda package, and decision curve analysis was performed to confirm its clinical utility.

### Subtyping OA based on coagulation

To investigate the potential of coagulation-related genes in stratifying osteoarthritis (OA), we performed unsupervised consensus clustering on OA samples. In the training set, clustering was conducted using the expression profiles of the two core genes (COL3A1 and MMP1) with the ConsensusClusterPlus R package, evaluating cluster numbers (K) from 2 to 9 to define preliminary subtypes. To assess the robustness of this classification approach, the same consensus clustering procedure (setting K = 2-9) was independently applied to the OA samples in the test set using the identical gene features. The resultant subtype labels from both cohorts were subsequently used as grouping variables for downstream comparative analyses, including immune microenvironment characterization.

### Enrichment analysis targeting biological functions

We identified differentially expressed genes (DEGs) for the new OA classification and performed enrichment analyses on these DEGs, including GSEA, KEGG, and GO analyses. A corrected p-value < 0.05 was used as the filtering criterion. This approach further elucidated the pathological mechanisms underlying stratified management of OA. We also performed subclustering based on the expression levels of the most critical core genes, employing the same methodology to explore the pathological mechanisms by which genes influence disease progression.

### Immune infiltration analysis

To comprehensively characterize the immune microenvironment across different OA subtypes, our analysis encompassed immune cell infiltration profiling and immune checkpoint gene expression assessment.

#### Immune cell infiltration profiling

We employed two complementary computational approaches to estimate immune cell abundance.

##### Single-sample gene set enrichment analysis

Immune cell enrichment scores were calculated using the gsva function from the GSVA R package (version 4.4.2) with the method=‘ssgsea’ parameter. A curated immune cell signature gene set collection (immune.gmt file) was used as the reference. The raw ssGSEA scores were normalized across samples using a min-max normalization procedure.

##### Cell-type deconvolution (CIBERSORT)

We estimated the relative fractions of 22 immune cell subtypes using the CIBERSORT.R script with the LM22 signature matrix (28). The analysis was performed with 1000 permutations and quantile normalization enabled (QN = TRUE). For robustness, immune cell subtypes estimated to have a non-zero fraction in less than 50% of all samples were filtered out from downstream visualizations.

#### Differential expression of immune checkpoint genes

A list of known immune checkpoint genes was curated from public databases (gene.txt). Genes expressed in our dataset were retained. Differential expression of these checkpoint genes between OA subtypes (defined by the risk group in our model) was assessed using the Wilcoxon rank-sum test. Genes with an adjusted P-value < 0.05 were considered significantly differentially expressed.

#### Visualization and integration

The relative abundances of infiltrating immune cell types (from CIBERSORT) were visualized in a heatmap using the pheatmap R package. To display an integrated landscape, a separate heatmap was generated using pheatmap to combine the normalized ssGSEA immune cell enrichment scores and the expression levels of differentially expressed immune checkpoint genes. Additional box plots and scatter plots were generated using the ggplot2 and ggpubr R packages.

### Isolation and culture of chondrocytes

For primary cell isolation, articular cartilage from 5-day-old wild-type mice was used in this study. All procedures were performed under sterile conditions in a biosafety cabinet. Using ophthalmic scissors, the separated cartilage was carefully cut into tiny tissue pieces on a sterile Petri plate. The fragments were pretreated for tissue by digesting them with a 0.25% trypsin solution while being constantly stirred(30 minutes, 37°C). This was followed by further enzymatic digestion in DMEM over six hours. Following two rounds of centrifugation to purify the isolated cells, the pellet was resuspended in full DMEM/F12 media containing 1% P/S with 10% FBS. After that, cell suspension was seeded into standard T25 flasks and kept in an incubator with humidity for primary growth (5% CO2, 37°C), changing the medium every 48 hours. To ensure functional stability, only passage 1 to passage 2 (P1-P2) cells were used in subsequent experiments. During subculturing, cell adherence and morphological traits were regularly observed.

### Using qRT-PCR technology for gene amplification and nucleic acid detection

The mouse COL3A1 overexpression vector (pCMV-COL3A1-Neo, pCOL3A1) and its negative control (pNC) were constructed by Tsingke Biotechnology. Plasmids were transfected into chondrocytes using LipoLeagene2000 according to the manufacturer’s instructions, with untreated cells serving as a blank control. To confirm successful overexpression, qRT-PCR was performed on cells from each experimental group. Total RNA was isolated under equal input conditions using the High-Purity RNA Isolation Kit and reverse-transcribed into cDNA using the High-Capacity cDNA Reverse Transcription Kit. The following primer sets were used for qRT-PCR:

• GAPDH (internal control):
 ○ Forward: *AGACAGCCGCATCTTCTTGT*
 ○ Reverse: *CTTGCCGTGGGTAGAGTCAT*
• COL3A1 (target gene):
 ○ Forward: *GCCTACATGGATCAGGCCAA*
 ○ Reverse: *CACCAGTGTGTTTAGTGCAGC*

### EdU assay for cell proliferation

To mimic an osteoarthritic microenvironment, chondrocytes from both the empty vector-transfected group (pNC) and the COL3A1-overexpressing group (pCOL3A1) were given 10 ng/mL of IL-1β for 12 hours after transfection, designated as pNC+IL-1β and pCOL3A1+IL-1β, respectively. These groups, along with the untreated pNC group, were subsequently subjected to further cellular assays. After equally seeding 6-well plates with the chondrocyte suspension, the plates were incubated for a full day at 37°C with 5% CO2. Following the removal of growth medium, we fixed the cells for ten minutes at room temperature using 4% paraformaldehyde (PFA), and then we washed them three times with PBS to get rid of any remaining fixative. In accordance with the EdU assay kit’s manufacturer’s instructions, cells were stained with DAPI solution for nuclear visualization after being incubated with the EdU reaction mixture while shielded from light. Finally, after washing the samples with PBS, we observed them at the fluorescence microscope. The assay was performed in three independent biological replicates, with three technical replicates per experiment. EdU-positive cells were counted in three randomly selected fields per well using ImageJ software. Data are presented as mean ± SD.

### Using JC-1 staining to assess mitochondrial membrane potential

Following their respective protocols, chondrocytes from each experimental group were seeded into 12-well plates and maintained in culture for 24 hours. To prepare for staining, the existing medium was removed, after which the cells in each well were exposed to pre-warmed JC-1 working solution (5 μM final concentration) and kept at 37°C in the absence of light for a 30-minute incubation period. Following the staining procedure, cells underwent three gentle washes with ice-cold PBS to eliminate any unbound dye. Visualization was carried out via an inverted fluorescence microscope configured for dual-channel acquisition: the red fluorescence from JC-1 aggregates was recorded with a 590 nm emission filter, and the green signal from monomers was captured with a 530 nm filter. The ratio of red-to-green fluorescence intensity was quantified using ImageJ software. Data were derived from three independent biological replicates.

### Alcian blue, Safranin O and Toluidine blue staining *in vitro*

To precisely identify cell surface chondroitin sulfate for Toluidine Blue staining, cells were treated with a 0.1% working solution over sixty minutes in a dark-controlled condition. Safranin O staining (0.02% working solution, 45 minutes) was employed to evaluate glycosaminoglycan distribution. Alcian Blue staining utilized a 1% solution (50-minute incubation) to visualize acidic mucopolysaccharide components. Following each staining step, we washed samples three times with PBS under gentle agitation (5 minutes per wash) to eliminate residual dye. Finally, we dehydrated specimens through a graded ethanol series, and we used inverted microscope to get broad-field pictures. Quantitative analysis of all images was performed under blinded conditions. The mean optical density (OD) was measured from three random fields per sample using ImageJ software. Data are expressed as the mean ± SD of at least three independent biological replicates (N = 3).

### Determination of reactive oxygen species

The DCFH-DA probe (Servicebio) was used to label cells in order to determine total intracellular ROS. The cell suspension was combined with the probe’s working solution (1:1000 dilution) and cultured over 30 minutes at 37°C in a light-protected environment. The unbound probe was then eliminated by rinsing it three times in HBSS buffer that had been heated beforehand. Fluorescence was visualized with the fluorescence microscope. Quantitative image analysis was performed by calculating the mean fluorescence intensity (MFI) with ImageJ software, with at least three biological replicates per treatment group included for statistical analysis. Quantitative image analysis was performed by calculating the mean fluorescence intensity using ImageJ software. Each treatment group included at least three biological replicates, with three technical replicate samples analyzed per experiment.

### Analysis of cellular senescence by SA-β-gal staining

In this study, we evaluated cellular senescence using β-galactosidase staining. Following 3 full days of treatment with βOHB and TBHP, a solution containing 2% glutaraldehyde and 0.2% formaldehyde was used to fix chondrocytes from various experimental groups over fifteen minutes at room temperature. After completing the above steps, cells were incubated with working solution (Beyotime) at 37°C for 16 hours under light-protected conditions. After staining, samples underwent three graded PBS washes. Senescence-associated β-galactosidase activity was visualized by detecting green precipitates. The percentage of SA-β-Gal-positive cells was counted in three randomly selected fields per sample using ImageJ software. The experiment was independently repeated three times (N = 3).

### Analysis of statistics

R (with 4.4.2 version) was used to conduct statistical analysis. All cell-based experiments included at least three biological replicates. We sourced the transcriptomic data from the Gene Expression Omnibus repository (GEO; https://www.ncbi.nlm.nih.gov/geo/). For comparisons across groups, a two-tailed Student’s t-test was applied, setting the significance level at P < 0.05. *, **, and *** indicate p-value <0.05, p-value <0.01, and p-value <0.001, respectively, and NS indicates no statistically significant difference. The sample size for each group was three, and the experiments for each sample were performed in three biological replicates.

## Results

### Examining of coagulation-related DEGs and exploration of their predictive value for OA

**Figure 1:**
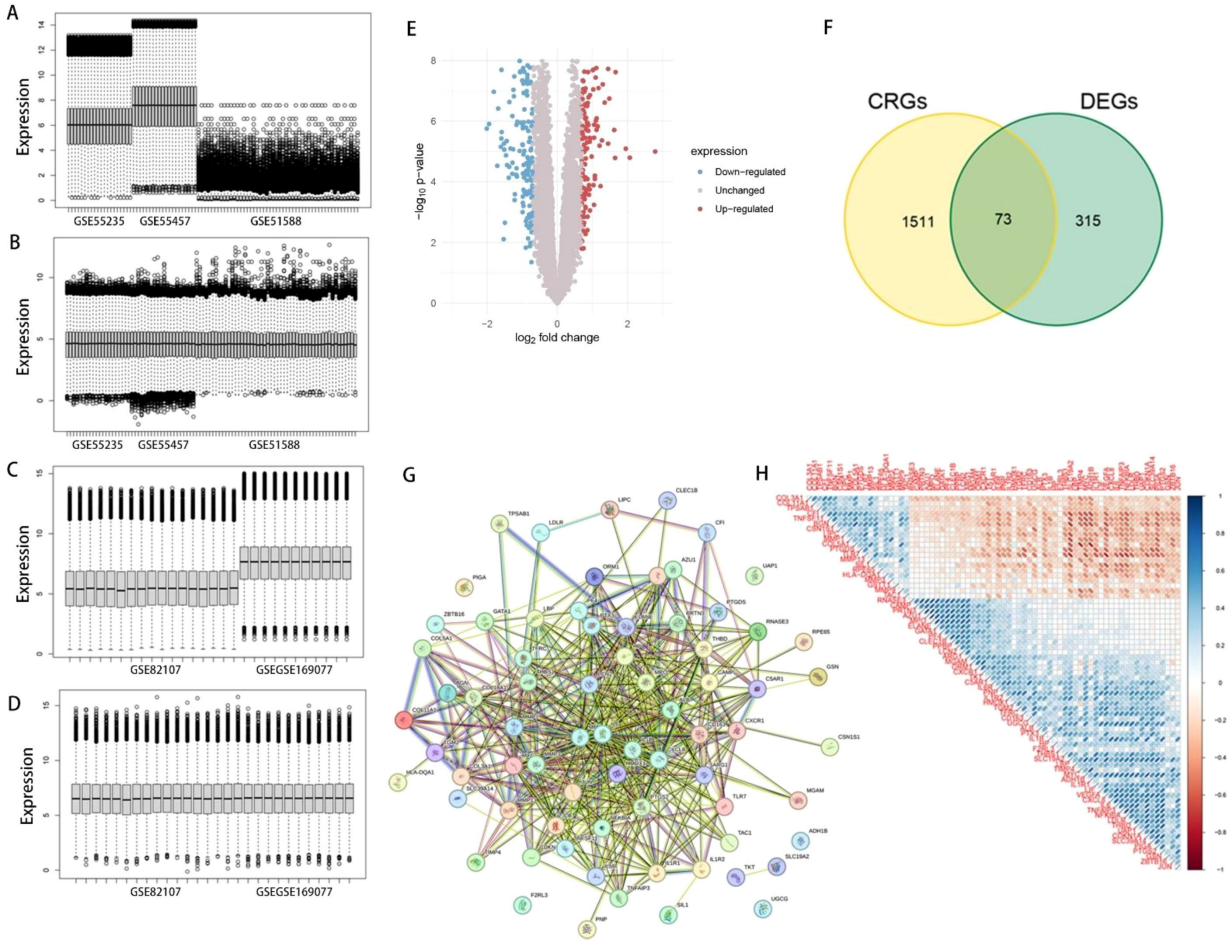
Screening process of DECRGs using GEO datasets. **(A, B)** Box plots display training set expression profiles prior to and following batch effect correction. **(C, D)** Test set expression distributions are shown before and after batch adjustment. **(E)** DEGs are visualized in a volcano plot. **(F)** Venn plot illustrating the overlapping genes identified from CRGs and DEGs. **(G)** PPI network of DECRGs. **(H)** Correlation heatmap displaying positive and negative correlations among DECRGs.

**Figure 2:**
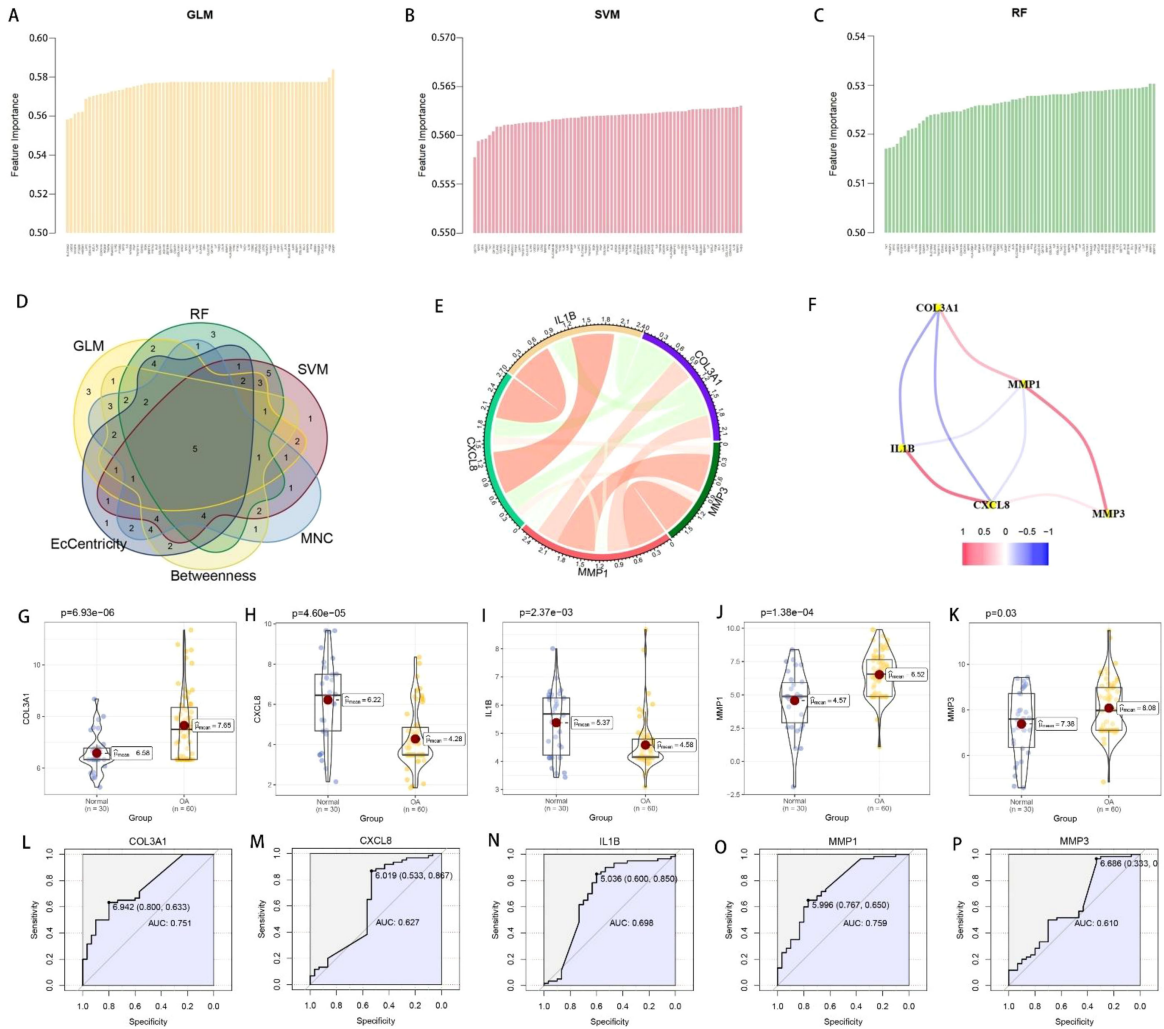
Further screening of DECRGs based on predictive value for OA. **(A–C)** Disease prediction value ranking of DECRGs using three machine learning methods: GLM, SVM, and RF. **(D)** Identification of five key genes (COL3A1, CXCL8, IL1B, MMP1, and MMP3) through multi-level screening with six distinct approaches. **(E)** A chord diagram visualizing correlation patterns among these 5 pivotal genes. **(F)** Node-link diagram illustrating correlations among the five key genes. **(G–K)** Violin plots showing differential expression of the five key genes. **(L–P)** The diagnostic capacity of the 5 pivotal genes for OA was assessed using ROC analysis.

**Figures 1A, B** display the expression box plots for the training set before or after removing batch effect, while **Figures 1C, D** show corresponding plots for the test set. We observed that batch effects were successfully removed in both datasets. **Figure 1E** presents the DEGs across different samples, genes with elevated expression in OA tissues are shown in red, while those more abundant in normal samples are depicted in blue. We identified 73 differentially expressed coagulation-related genes (DECRGs) by taking the intersection CRGs and DEGs, as shown in **Figure 1F**. A protein-protein interaction (PPI) network for these 73 DECRGs was constructed and is displayed in **Figure 1G**. The positive or negative correlation relationships among these DECRGs are illustrated in **Figure 1H**. To evaluate the disease-predictive potential of the 73 DECRGs, we employed three machine learning methodologies: RF, SVM, and GLM. The results of the feature importance ranking from these methods are shown in **Figures 2A–C**, respectively. Additionally, we calculated and ranked the DECRGs based on EcCentricity, Betweenness, and MNC values. By taking the intersection of the top 50% of genes from all six ranking methods, we identified five key genes crucial for OA: COL3A1, CXCL8, IL1B, MMP1, and MMP3, as summarized in **Figure 2D**. The correlation relationships among these five key genes are visualized in a correlation chord diagram (**Figure 2E**) and a node-link diagram (**Figure 2F**). To further explore the disease predictive value of these key genes, we generated violin plots comparing their expression levels of OA samples with healthy control samples. As displayed in **Figures 2G–K**, OA samples exhibited markedly elevated expression levels of COL3A1, MMP1, and MMP3 relative to healthy controls. We plotted ROC for evaluating the independent diagnostic accuracy of each of the five key genes for OA. The results are presented in **Figures 2L** through 2P. Both COL3A1 and MMP1 demonstrated discriminative ability, with AUC values exceeding 0.7, suggesting that COL3A1 and MMP1 hold superior diagnostic potential for OA among these evaluated gene candidates.

### OA subtyping and diagnosis based on key genes COL3A1 and MMP1

**Figure 3:**
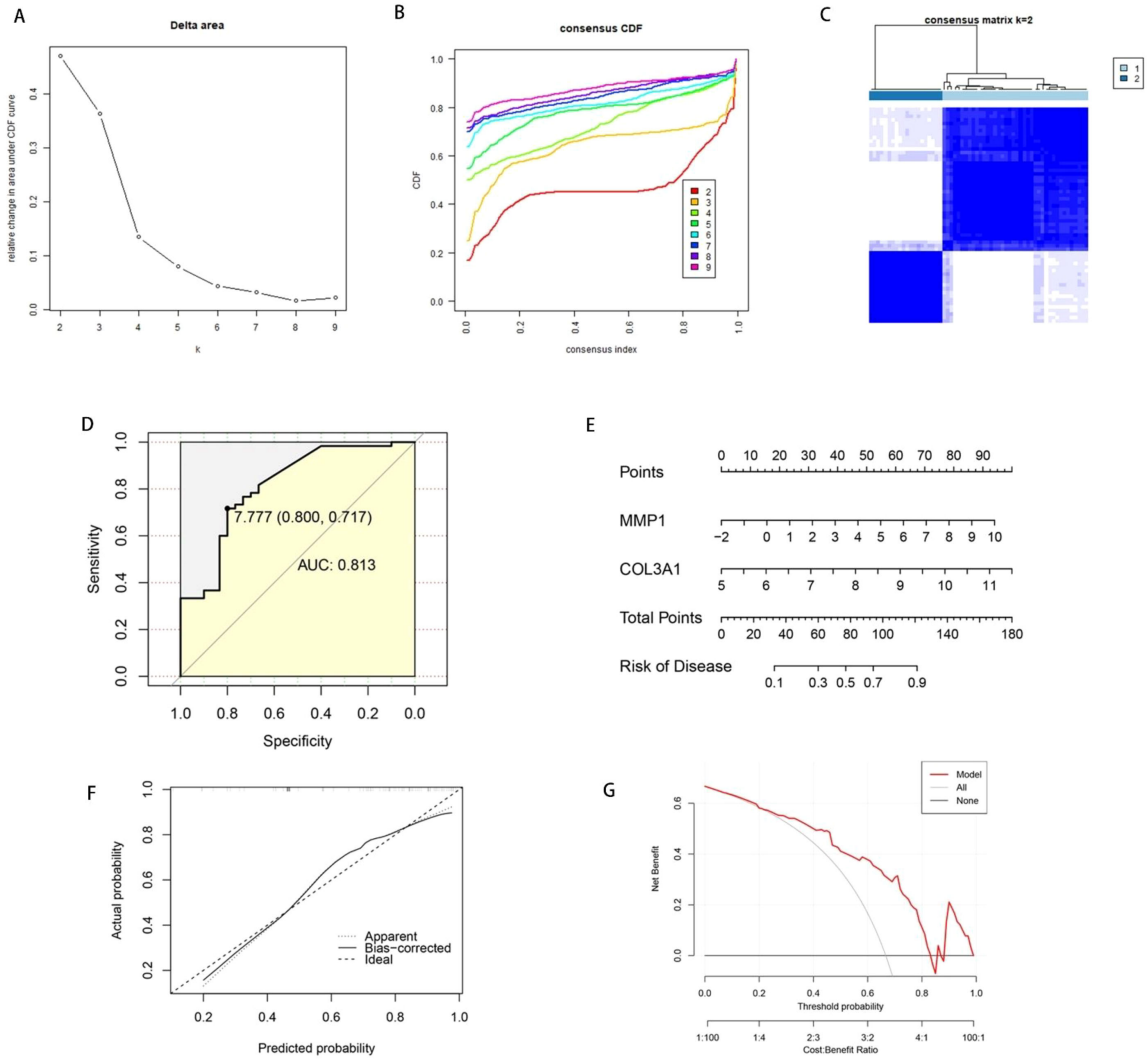
OA subtyping based on COL3A1 and MMP1 and construction of nomogram. **(A–C)** Optimal clustering performance for OA samples from the training cohort was achieved at cluster number K = 2. **(D)** Diagnostic performance of the OA model in the training cohort as evaluated by ROC analysis. **(E)** Nomogram constructed based on COL3A1 and MMP1 expression levels. **(F)** Calibration curve of the nomogram. **(G)** DCA of the nomogram.

**Figure 4:**
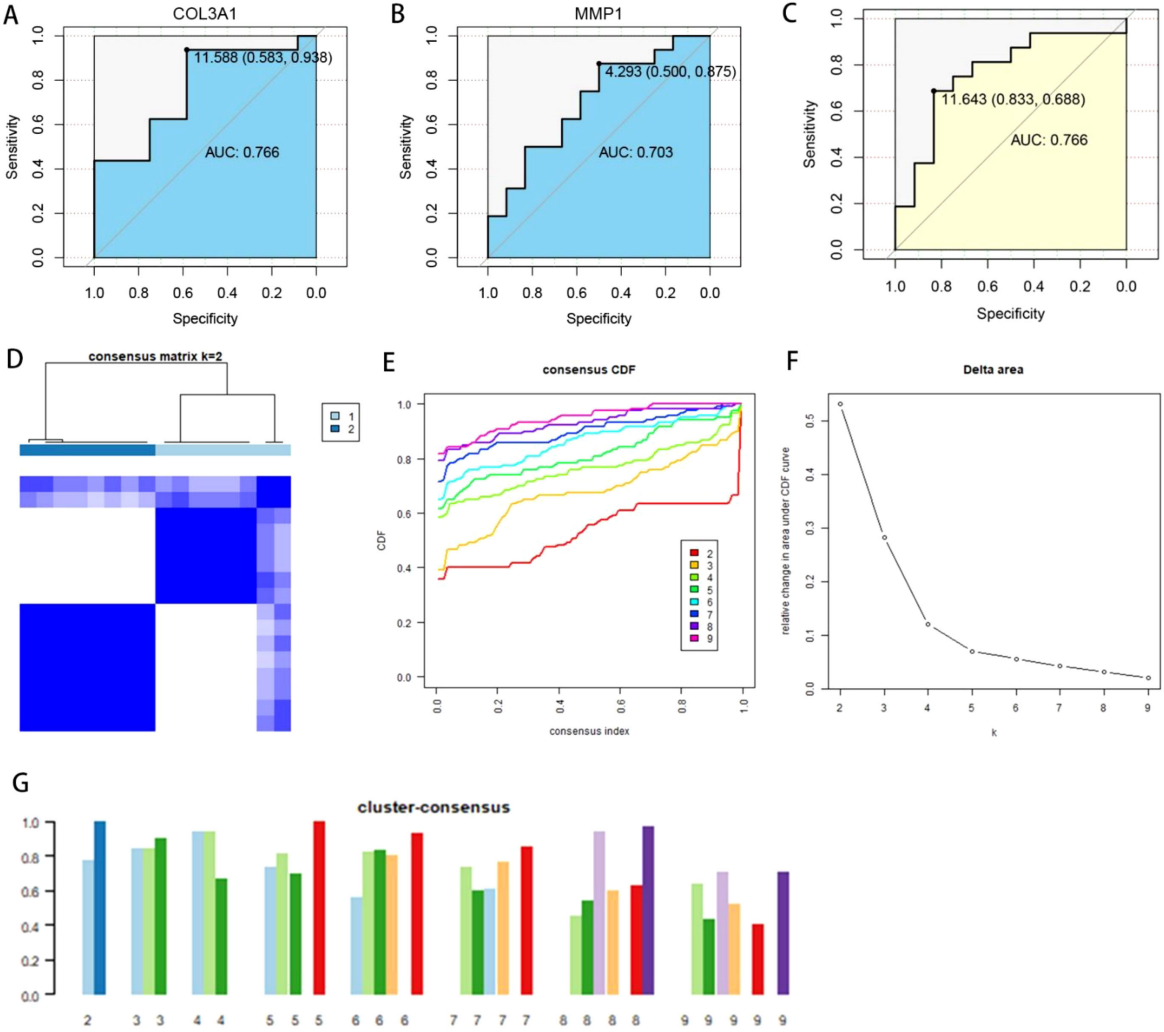
Test cohort evaluation: verification of OA subtypes and diagnostic model performance. **(A)** ROC curve for COL3A1 in the test set. **(B)** Test cohort ROC analysis for MMP1. **(C)** Diagnostic model performance evaluation in the test cohort. **(D–G)** Unsupervised clustering performance based on COL3A1 and MMP1 expression in the test set.

We utilized COL3A1 and MMP1, which had the highest AUC values, to perform multivariate logistic regression for constructing an OA diagnostic model. The model is represented as: Riskscore = (MMP1 expression × 0.3893437) + (COL3A1 expression × 0.7646114). Each sample was assigned a risk score estimating its probability of having OA. For the training cohort, a risk score-based ROC curve was developed to assess OA predictive accuracy. This model demonstrated strong discriminative power with an AUC of 0.813, validating its diagnostic reliability. Unsupervised clustering was applied to training set OA samples, utilizing expression patterns of MMP1 and COL3A1 for classification. Our results indicated optimal clustering performance when the cluster number (K) was set to 2, as shown in **Figures 3A–C**. Additionally, we built a nomogram for OA prediction according to COL3A1 and MMP1 expression (**Figure 3E**). The calibration curve (**Figure 3F**) and DCA (**Figure 3G**) of the nomogram both demonstrated its high predictive potential. To further confirm the stability of our established OA subtyping and diagnostic model, we evaluated it in the test set. ROC curves were plotted for the individual risk genes (COL3A1 and MMP1) and the combined diagnostic model, yielding AUC of 0.766, 0.703, 0.766 (**Figures 4A–C**). Furthermore, clustering based on COL3A1 and MMP1 expression in the test set also showed excellent performance, as presented in **Figures 4D–G**.

### Exploring potential biological functional differences between OA subtypes

**Figure 5:**
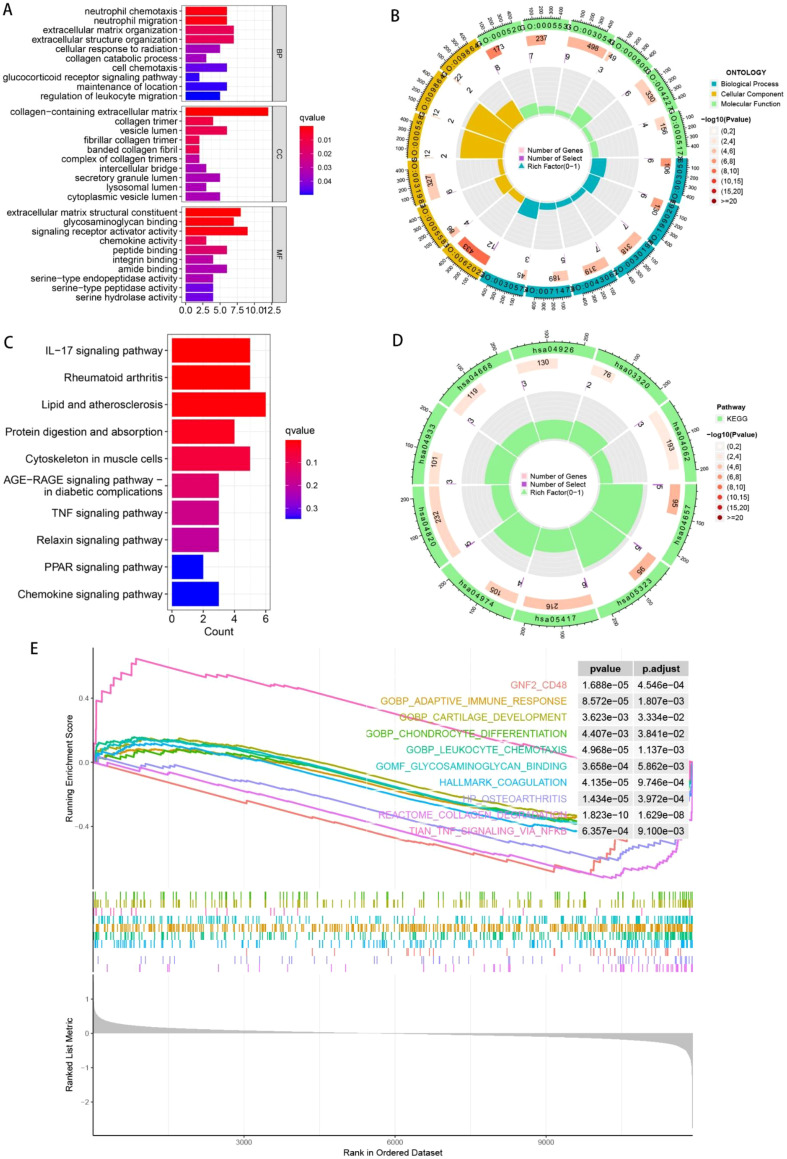
Using enrichment analysis, biological activities and molecular pathway variations among various OA subtypes are investigated. **(A)** Results of GO enrichment analysis of DEGs among different OA subtypes. Bar graphs display the outcomes of gene enrichment in MF, CC, and BP. **(B)** GO enrichment analysis findings are displayed in a circle diagram. **(C, D)** KEGG enrichment analysis results. **(E)** GSEA enrichment analysis results.

Differential expression analysis was conducted between C1 and C2 OA subtypes to elucidate their distinct pathological mechanisms and evaluate variations in biological functions and molecular pathways, we also performed further enrichment analysis on these DEGs. The enrichment results of DEGs in MF, CC, and BP are displayed in **Figure 5A**. These genes showed significant enrichment in pathways associated with cartilage degeneration including collagen catabolic process, extracellular matrix containing collagen, collagen trimer, fibrillar collagen trimer, glycosaminoglycan binding, peptide binding, and serine hydrolase activity. Additionally, GO analysis revealed immune-related pathways such as neutrophil chemotaxis, leukocyte migration regulation, and neutrophil migration. In **Figure 5B**, the GO findings are displayed as circle diagrams. KEGG enrichment results are shown in **Figures 5C, D**, and the genes were significantly enriched in protein digestion and absorption, Rheumatoid arthritis, IL-17 signaling pathway, Lipid and atherosclerosis, TNF, AGE-RAGE, Relaxin, PPAR, Chemokine signaling pathway. With reference to the GSEA enrichment analysis, on the other hand, indicated that these genes appeared substantially abundant in pathways including GNF2 CD48, adaptive immune response, cartilage development, chondrocyte differentiation, leukocyte chemotaxis, glycosaminoglycan binding, coagulation, osteoarthritis, collagen degradation, TNF signaling via NFKB, as shown in **Figure 5E**. These pathways are not only associated with coagulation and osteoarthritis development, but also contain immune infiltration-related pathways. As a result, we postulated that there are variations in immune infiltration among various subtypes, which merits further investigation as a treatment approach for osteoarthritis.

### Investigating the immunological milieu of several subtypes of OA

**Figure 6:**
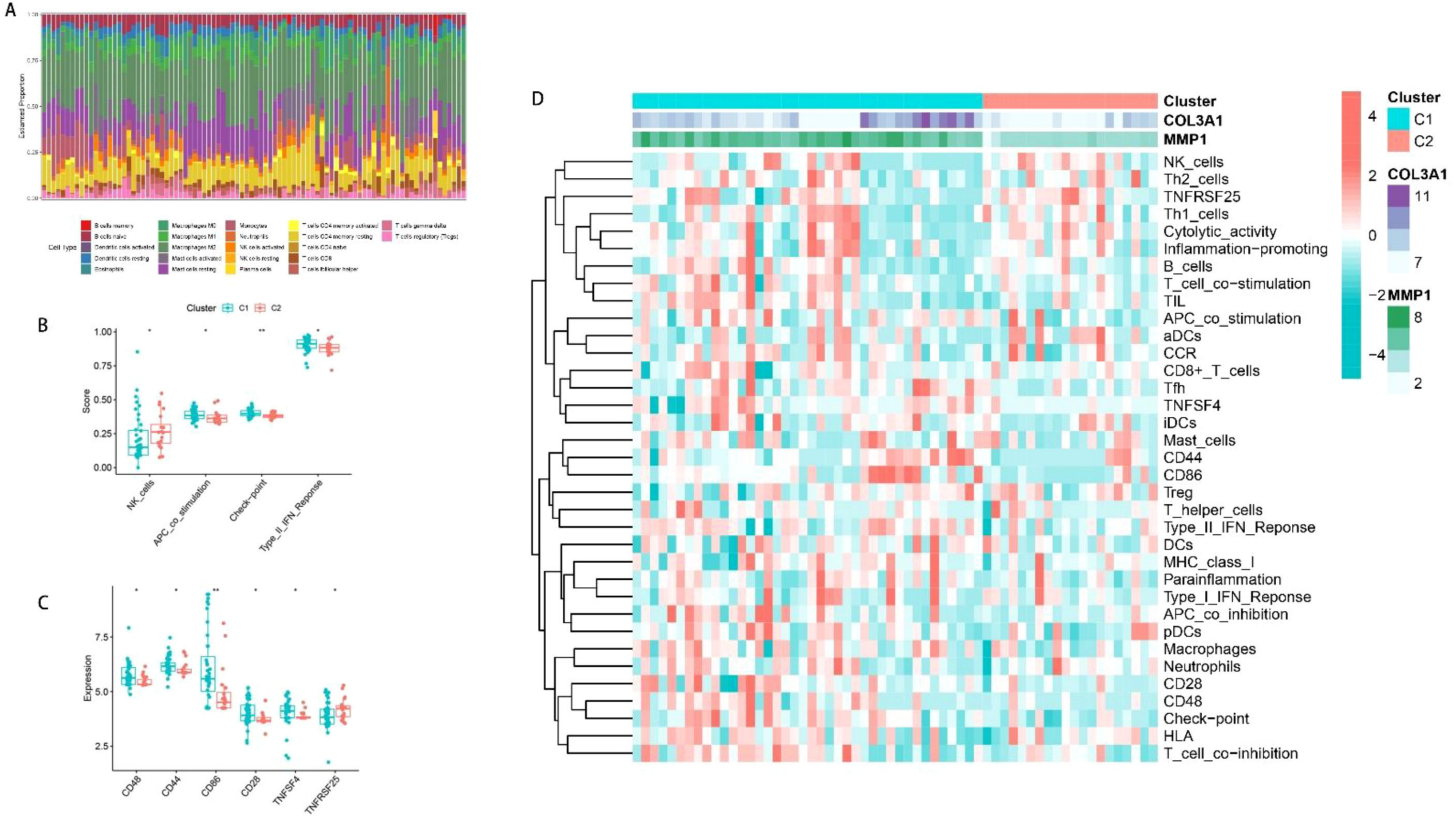
Immune profiling of osteoarthritis subtypes. **(A)** Normalized CIBERSORT immune cell distributions visualized using box-line plots. **(B)** Notable variations were observed in NK cell activity, type II interferon signaling, immune checkpoint markers, and antigen-presenting cell co-stimulation across OA subtypes. **(C)** Immune checkpoint expression levels (CD28, CD44, CD48, CD86, TNFSF4, TNFRSF25) showed substantial variation between OA subtypes. **(D)** Immunoinfiltration heat map of different OA subtypes.

Enrichment results indicated potential immune pathway variations across OA subtypes. Subsequent CIBERSORT and immune infiltration analyses were therefore conducted to characterize these immunological differences. Immune infiltration profiling across all samples (**Figure 6A**) revealed macrophages as the predominant immune cell population. Subtype comparisons in **Figure 6B** demonstrated markedly distinct immune activity profiles, including NK cell infiltration, antigen-presenting cell co-stimulation, type II interferon responses, and immune checkpoint expression. **Figure 6C** revealed substantial disparities in immune checkpoint expression (CD48, CD44, CD86, CD28, TNFSF4, TNFRSF25) across OA subtypes. **Figure 6D** demonstrated distinct immune infiltration patterns between C1 and C2 subtypes, with C1 exhibiting moderately elevated immune infiltration levels and significantly higher expression of both COL3A1 and MMP1 compared to C2.

### Investigating relationships between pivotal genes and immune cell infiltration

**Figure 7:**
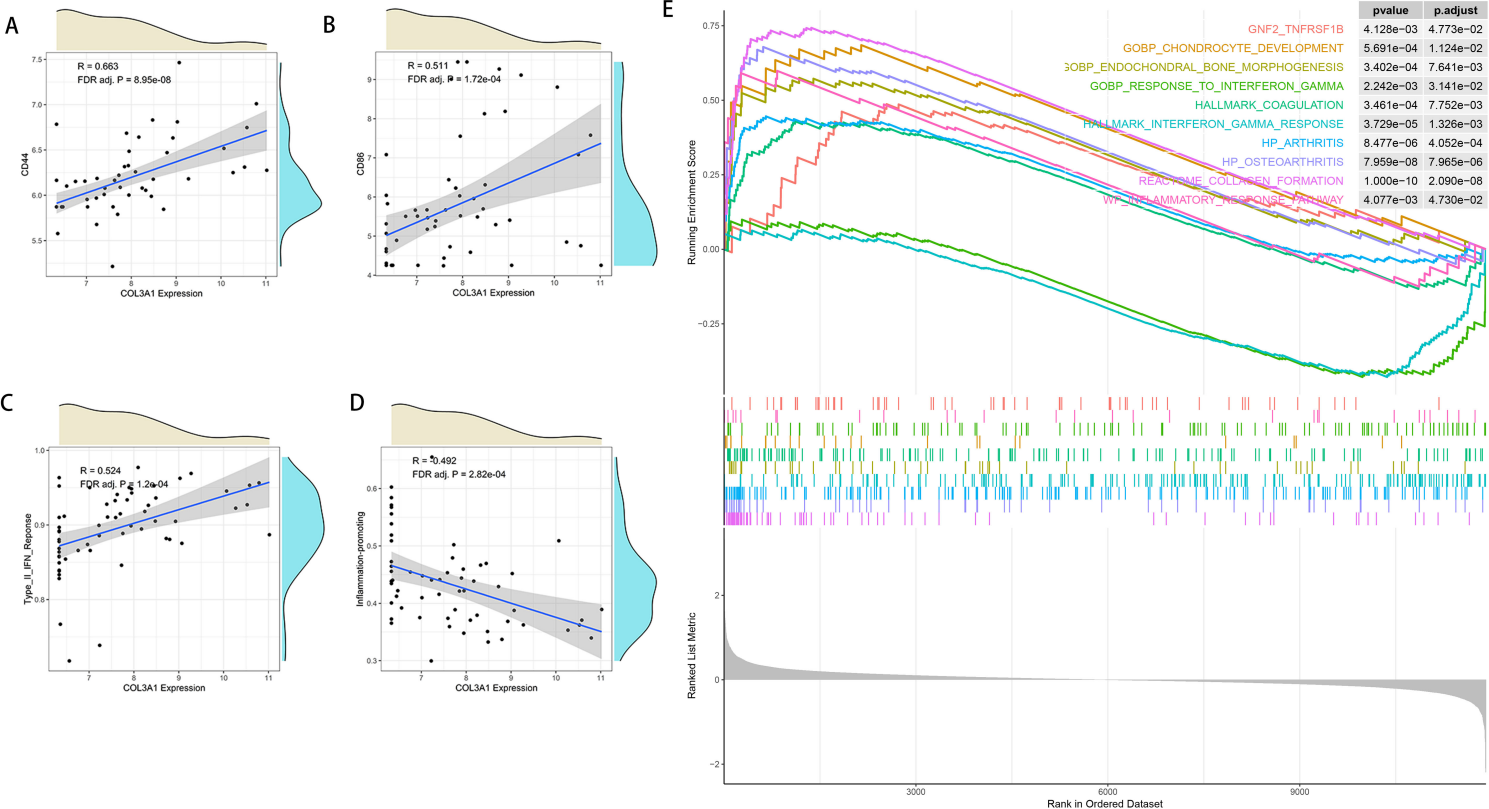
Associations between COL3A1 expression and immune infiltration features. **(A–D)** Scatter plots showing the correlations between COL3A1 expression and immune features that remained statistically significant after false discovery rate (FDR) correction: positive correlations with CD44 **(A)**, CD86 **(B)**, and Type II IFN response **(C)**; and a negative correlation with Inflammation-promoting **(D)**. P-values are FDR-adjusted. **(E)** COL3A1 was significantly and positively correlated with GNF2 TNFRSF1B, Chondrocyte development, endochondrol bone morphogenesis, response to interferon gamma, coagulation, interferon gamma response, arthritis, osteoarthritis, collagen formation, and inflammatory response pathway.

We next examined how pivotal genes correlate with immune infiltration patterns in OA. We selected COL3A1, which had a higher risk coefficient (0.7646114) and a higher AUC value (0.766) in the test set, for subsequent analysis. After applying Benjamini-Hochberg false discovery rate (FDR) correction for multiple testing, COL3A1 expression levels showed significant positive correlations with CD44, CD86, and Type II IFN response, and a significant negative correlation with Inflammation-promoting (FDR-adjusted P < 0.05; **Figures 7A–D**). These results suggest that COL3A1 is associated with a distinct immune-inflamed phenotype. This finding is consistent with our observation that COL3A1 expression was substantially elevated in the C1 OA subtype, which also demonstrated markedly higher levels of immune infiltration compared to the C2 subtype. **Figure 7E** demonstrated the GSEA enrichment results of COL3A1, which showed that COL3A1 was associated with pathways including GNF2 TNFRSF1B, chondrocyte development, endochondral bone morphogenesis, response to interferon gamma, coagulation, interferon gamma response, arthritis, osteoarthritis, collagen formation, and inflammatory response. These pathways are collectively associated with osteoarthritis progression, coagulation, and immunity, suggesting that COL3A1 may affect OA progression by regulating biological processes such as chondrocyte development, subchondral bone formation, and immune-metabolic crosstalk.

### COL3A1 overexpression suppresses chondrocyte proliferation and matrix synthesis

**Figure 8:**
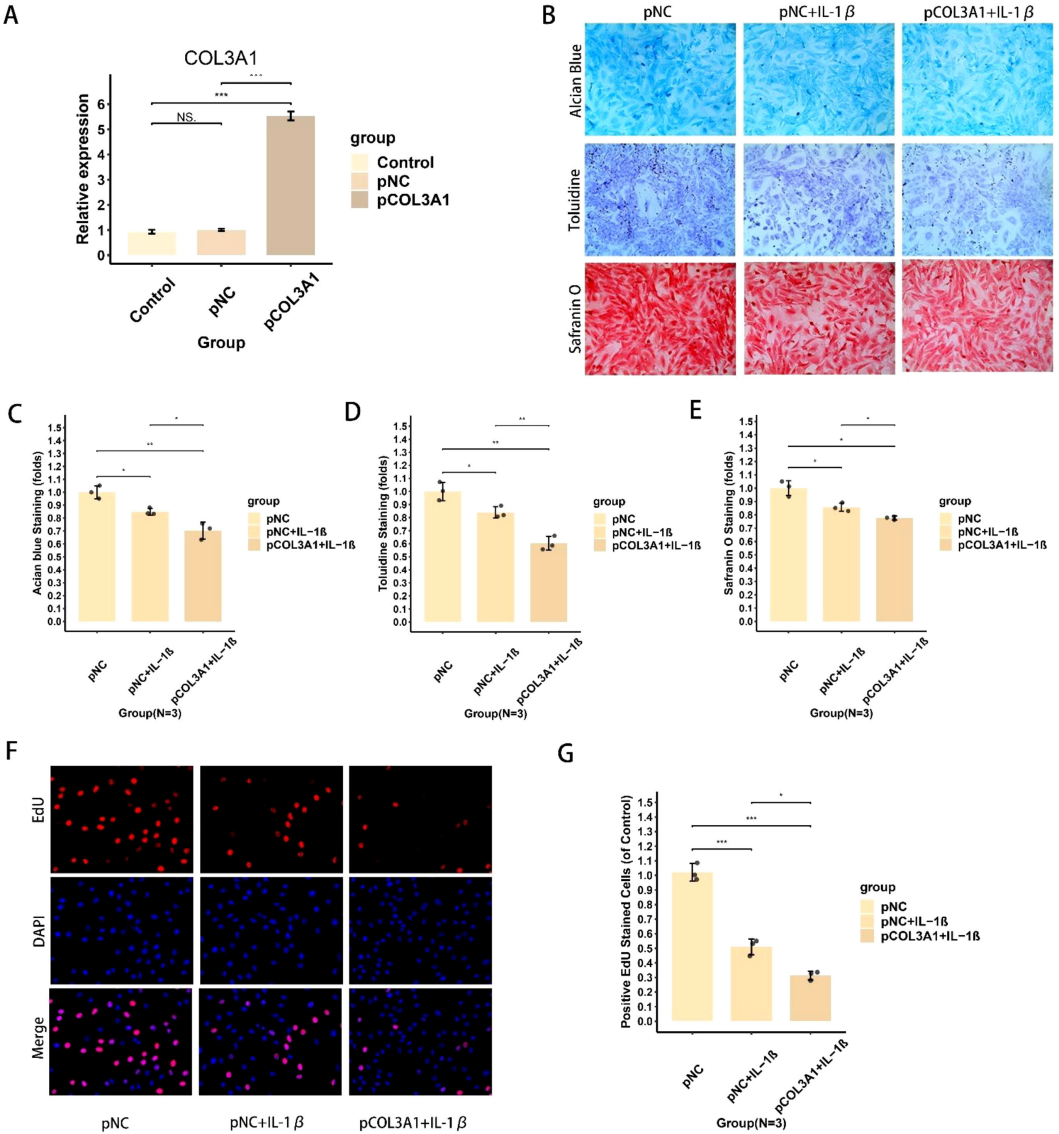
Effects of COL3A1 overexpression on chondrocyte proliferation and extracellular matrix anabolism. **(A)** Validation of successful COL3A1 overexpression in chondrocytes via PCR analysis. **(B)** Representative images of cytochemical staining (Safranin O, Toluidine Blue, Alcian Blue) for evaluating the content of proteoglycans and glycosaminoglycans. **(C–E)** Quantitative analysis of the staining intensity for Alcian Blue **(C)**, Toluidine Blue **(D)**, and Safranin O **(E)**, demonstrating the impairment of extracellular matrix synthesis. **(F)** Representative images of EdU staining assessing chondrocyte proliferation. **(G)** Quantitative analysis of chondrocyte proliferation rates based on EdU staining. *, **, and *** indicate p-value <0.05, p-value <0.01, and p-value <0.001, respectively, and NS indicates no statistically significant difference. The sample size for each group was three, and the experiments for each sample were performed in three biological replicates.

To investigate the impact of COL3A1 on the chondrocyte phenotype, we established a COL3A1-overexpressing chondrocyte model (pCOL3A1) and a control (pNC) via plasmid transfection, with successful overexpression confirmed by PCR (**Figure 8A**). Subsequently, both pNC and pCOL3A1 cells were treated with IL-1β to simulate the osteoarthritic inflammatory environment. Cytochemical staining with Safranin O, Toluidine Blue, and Alcian Blue demonstrated that IL-1β stimulation markedly reduced the content of glycosaminoglycans and proteoglycans, indicating impaired extracellular matrix synthesis (**Figure 8B**). Quantitative analysis confirmed that this IL-1β-induced impairment was significantly exacerbated by COL3A1 overexpression (**Figures 8C–E**). Furthermore, the EdU assay revealed that IL-1β inhibited chondrocyte proliferation, an effect that was further enhanced in COL3A1-overexpressing cells, as shown by representative images (**Figure 8F**) and quantitative analysis (**Figure 8G**). Collectively, these results indicate that COL3A1 overexpression suppresses both the anabolic function and proliferative capacity of chondrocytes under inflammatory OA conditions.

### COL3A1 exacerbates mitochondrial dysfunction and senescence in OA chondrocytes

**Figure 9:**
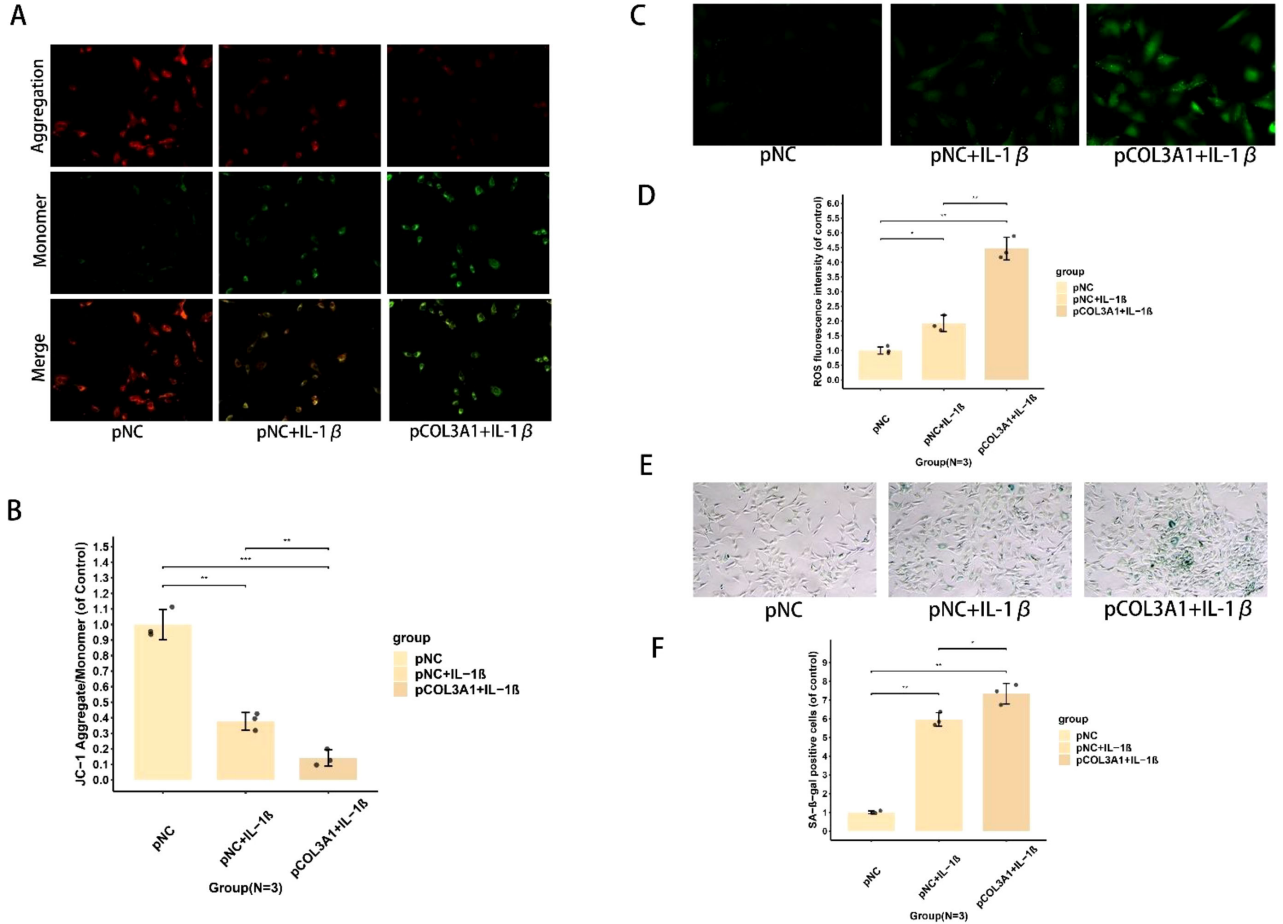
COL3A1 overexpression exacerbates mitochondrial dysfunction, oxidative stress, and cellular senescence in chondrocytes under osteoarthritic conditions. **(A)** Representative images of JC-1 staining for analyzing mitochondrial membrane potential. **(B)** Quantitative analysis of mitochondrial membrane potential based on JC-1 staining. **(C)** Representative images showing intracellular ROS levels. **(D)** Quantitative measurement of intracellular ROS levels. **(E)** Representative images of SA-β-gal staining for assessing chondrocyte senescence. **(F)** Quantitative analysis of SA-β-gal positive cells. *, **, and *** indicate p-value <0.05, p-value <0.01, and p-value <0.001, respectively, and NS indicates no statistically significant difference. The sample size for each group was three, and the experiments for each sample were performed in three biological replicates.

To further elucidate the mechanisms by which COL3A1 affects chondrocyte function, we assessed mitochondrial function, oxidative stress, and senescence-related markers. Analysis of mitochondrial membrane potential via JC-1 staining revealed that IL-1β treatment led to a marked decrease in potential, an effect that was significantly exacerbated by COL3A1 overexpression, as shown in representative images (**Figure 9A**) and confirmed by quantitative analysis (**Figure 9B**). Similarly, measurement of intracellular reactive oxygen species (ROS) levels demonstrated that IL-1β stimulation significantly elevated oxidative stress, and COL3A1 overexpression further amplified this ROS production (**Figures 9C, D**). Finally, assessment of cellular senescence via SA-β-gal staining confirmed that IL-1β promoted chondrocyte senescence, a pro-senescent effect that was markedly enhanced by COL3A1 overexpression (**Figures 9E, F**). In summary, these results demonstrate that under osteoarthritic conditions, COL3A1 overexpression exacerbates chondrocyte degeneration by impairing mitochondrial function, intensifying oxidative stress, and promoting cellular senescence.

## Discussion

Despite advances in understanding osteoarthritis (OA), its pathological heterogeneity and the complex interplay of networks such as inflammation and tissue remodeling remain central challenges (29). Within this context, the role of the coagulation system, particularly its cross-talk with inflammation and fibrosis (the coagulation-inflammation-fibrosis axis), has emerged as a relevant but underexplored area in OA pathogenesis (30–34). To systematically explore this axis, we adopted a broad bioinformatics approach to identify and characterize key “coagulation-related” molecular players in OA. The following sections interpret our stepwise findings, from biomarker discovery to functional validation, and discuss their implications.

Five osteoarthritis and normal control datasets were acquired from the GEO repository. These datasets were randomly allocated into training and test cohorts, with subsequent analyses performed following batch effect correction. We acquired coagulation-related genes from the GeneCards database and identified 73 DECRGs through intersection with OA-derived DEGs. Correlation heatmap showed significant positive or negative correlation between DECRGs, suggesting the presence of co-expression patterns. Protein interaction network maps showed that the DECRGs were heavily regulated with each other. Three machine learning methods GLM, RF, and SVM were used to rank the importance of DECRGs, and Bewteenness, MNC, and EcCentricity values were used to further screen the DECRGs. Subsequently, the intersection was taken to obtain the five core genes COL3A1, CXCL8, IL1B, MMP1, and MMP3, and the correlation node network graphs and the correlation chord plot showed the relationship between the core genes, in which COL3A1, MMP1 and MMP3 were highly expressed in OA samples. MMP1 and COL3A1 demonstrated strong diagnostic capability for OA, with AUC values of 0.759 and 0.751 respectively. A combined diagnostic model incorporating both genes achieved an AUC of 0.813, confirming its robust performance. And the predictive nomogram developed using MMP1 and COL3A1 reliably forecasted osteoarthritis occurrence. Subsequently, cluster analysis utilizing MMP1 and COL3A1 expression patterns distinctly stratified OA samples into C1 and C2 subtypes, offering novel perspectives for patient stratification and personalized therapeutic approaches. Similarly, we performed diagnostic model construction, cluster analysis, and ROC construction for single-gene prediction of disease in the test set, and the results were still very impressive. In the test set, the AUC values of COL3A1 and MMP1 for diagnosing OA were as high as 0.766 and 0.703, and that of the diagnostic model was as high as 0.766. The OA samples were also able to be clustered into two classes, which validate the strong translational potential of both the diagnostic model and core genes.

Then, differential expression analysis was conducted between OA subtypes to elucidate their distinct pathological mechanisms, and obtained the differentially expressed genes followed by enrichment analysis. GO enrichment analysis showed that these genes were enriched in biological pathways related to cartilage degeneration, such as collagen catabolic process, collagen-containing extracellular matrix, collagen trimer, fibrillar collagen trimer, glycosaminoglycan binding, peptide binding, serine hydrolase activity, and also immune-related pathways, such as neutrophil chemotaxis, neutrophil migration, regulation of leukocyte migration, etc. KEGG enrichment analysis showed that the genes were enriched in pathways such as IL-17 signaling pathway, Rheumatoid arthritis, Lipid and atherosclerosis, protein digestion and absorption, Cytoskeleton in muscle cells, AGE-RAGE signaling pathway in diabetic complications, TNF signaling pathway, Relaxin signaling pathway, PPAR signaling pathway, Chemokine signaling pathway. GSEA enrichment analysis yielded pathways such as GNF2 CD48, adaptive immune response, cartilage development, chondrocyte differentiation, leukocyte chemotaxis, glycosaminoglycan binding, coagulation, osteoarthritis, collagen degradation, TNF signaling via NFKB. These pathways are not only associated with coagulation and osteoarthritis development, but also contain immune infiltration-related pathways. We consequently postulated that immune infiltration patterns vary across OA subtypes, warranting in-depth investigation as potential therapeutic targets. Immune infiltration analysis revealed the highest percentage of macrophages in all samples. There were significant differences in NK cells, APC co-stimulation, check point, and Type II IFN response between the different OA subtypes, and immune checkpoint levels CD48, CD44, CD86, CD28, and TNFSF4, TNFRSF25 also differed significantly. Overall, the degree of immune infiltration was slightly higher in C1 than in C2, and the expression of both COL3A1 and MMP1 was higher in the C1 subtype than in the C2 subtype, suggesting that COL3A1 and MMP1 may promote immune infiltration in OA. We selected COL3A1 with a higher risk coefficient (0.7646114) as well as a higher AUC value (0.766) in the test set for subsequent analysis, and found that COL3A1 expression levels were significantly positively correlated with CD44, CD86, TNFSF4, and Type II IFN response, whereas they were significantly negatively correlated with Type I IFN response and inflammation promoting. These results suggested that COL3A1 may increase immune infiltration, especially immune checkpoints, which is consistent with the high expression of COL3A1 and higher level of infiltration in the C1 subtype. Lu et al (35) indicated that the level of CD86 + B cells increased with the increase in the severity of osteoarthritis. Li et al (36) found increased expression of CD44 in damaged articular cartilage of mice in animal experiments, and a significant increase in CD44 levels was also found in synovial fluid samples from humans with osteoarthritis. And this study can also infer that COL3A1 may promote the progression of OA through these markers.

GSEA enrichment of COL3A1 showed that COL3A1 was significantly associated with GNF2 TNFRSF1B, Chondrocyte development, endochondrol bone morphogenesis, response to interferon gamma, coagulation, interferon gamma response, arthritis, osteoarthritis, collagen formation and inflammatory response pathway. These pathways are associated with osteoarthritis progression, coagulation, and immunity, suggesting that COL3A1 may affect osteoarthritis progression by regulating chondrocyte development, subchondral bone formation and coagulation. The COL3A1 gene encodes the alpha1 chain of type III collagen, which is an important fibrillar collagen in the extracellular matrix (ECM), and it is mainly distributed in the elastic tissues of the vascular wall, and is responsible for maintaining the structural integrity and mechanical stability of tissues (37, 38).Type III collagen regulates the flexibility and tensile strength of the ECM by co-polymerizing with type I collagen to form heterogeneous fibers, and participates in processes such as cell migration, differentiation and tissue repair (39). In recent years, abnormal COL3A1 expression has been found to be associated with a variety of diseases, including fibrotic diseases, tumor microenvironmental remodeling, and degenerative joint pathologies (40–42). It was found that COL3A1 expression was significantly elevated in subchondral bone tissues of OA patients, suggesting that it may be involved in OA progression through the regulation of ECM metabolism (43). In addition, COL3A1 is co-expressed with matrix metalloproteinases (MMPs) and members of the ADAMTS family, which may accelerate the degradation of cartilage matrix through activation of MMPs, thereby exacerbating joint damage (43). Chen et al (44) found that COL3A1 can affect chondrocyte function and is a diagnostic marker for patients with primary SC. While COL3A1 research in OA remains limited, our investigation firstly identified its independent role in OA pathogenesis, established connections between COL3A1, immune infiltration and osteoarthritis progression, and successfully classified OA subtypes using COL3A1 and MMP1 expression profiles. COL3A1 and MMP1, although not classical coagulation factors, are mechanistically related to coagulation pathology: COL3A1 is integral to the vascular matrix upon which coagulation initiation is dependent (19, 20), while MMP1 is a known activator of the coagulation-related PAR1 signaling pathway (21).

Subsequently, in this study, the basic experiment showed that IL-1β-treated chondrocytes showed a significant decrease in proliferative capacity, cell viability, and mitochondrial function, and a significant increase in the proportion of damaged and senescent chondrocytes, which was further strengthened by COL3A1 overexpression. Our findings demonstrate that COL3A1 overexpression exacerbates chondrocyte damage and cell death within the osteoarthritic milieu, thereby accelerating disease progression. Collectively, we developed a reliable OA risk signature applicable for subtype stratification, immune infiltration assessment, and clinical diagnosis. Our bioinformatics analysis and basic experiments also confirmed the significant effect of COL3A1 on chondrocytes in the osteoarthritic environment, which should be emphasized as an important intervention target in osteoarthritis, and it is of great significance for osteoarthritis monitoring and treatment.

This study has several limitations. First, as a retrospective analysis utilizing public databases, we were unable to control for potential confounding clinical variables (e.g., detailed OA severity, precise anatomical subsite, comprehensive patient demographics). This may introduce unmeasured heterogeneity; therefore, future validation of our findings in prospective cohorts with well-characterized clinical phenotypes is warranted. Second, the integration of cartilage and synovial tissue data in the bioinformatic analysis inevitably introduced tissue heterogeneity. This particularly complicates the cellular interpretation of immune infiltration patterns (e.g., the proportion of M0 macrophages), although it may also reflect common pathological events across tissues, such as “coagulation-immune crosstalk.” Third, the OA subtypes defined based on transcriptomic profiles require validation in independent, prospectively collected clinical cohorts with complete phenotypic data to establish their clinical relevance and stability. Fourth, while the *in vitro* functional validation confirmed the exacerbating role of COL3A1 in IL-1β-induced chondrocyte injury, a simplified model (single inflammatory stimulus) was used. Future studies employing loss-of-function experiments (e.g., COL3A1 knockdown) are needed to establish causality. Fifth, although the two-gene model was able to stratify OA subtypes and predict the disease, the combined diagnostic model did not show a statistically significant improvement in performance compared to the COL3A1 single-gene model in the test set. Finally, the associations between COL3A1 and specific immune features remain correlative, and their underlying mechanisms warrant further investigation. Despite these limitations, our core findings—establishing COL3A1 as a stable biomarker and a functional regulator in OA chondrocytes, and proposing a novel molecular subtyping strategy—provide valuable hypotheses and targets for future research.

## Conclusion

This study elucidates the key role of the coagulation pathway in the pathogenesis of OA and illuminates the regulatory function of COL3A1 on chondrocyte response in the inflammatory microenvironment, providing new perspectives for the diagnosis and treatment strategies of OA.

## Data Availability

The original contributions presented in the study are included in the article/**Supplementary Material**. Further inquiries can be directed to the corresponding authors.

## Supplementary material

The Supplementary Material for this article can be found onlineat: https://www.frontiersin.org/articles/10.3389/fimmu.2026.1759281/full#supplementary-material
