# Supplementary material for: Coagulation-related genes COL3A1 and MMP1 influence the development of osteoarthritis and the surrounding immunological environment
Source: Front Immunol. 2026 Jan 28;17:1759281. doi: 10.3389/fimmu.2026.1759281 (PMC12890649; doi:10.3389/fimmu.2026.1759281)
Supplement: Supplementary file 1 [file Table1.docx]

**Supplementary Tables**Table S1. DeLong test for ROC curves of COL3A1, MMP1, and the prediction model in the training set.

| Comparison | AUC1 | AUC2 | Z | P value | Adjust-p value |
| --- | --- | --- | --- | --- | --- |
| COL3A1 vs MMP1 | 0.751 | 0.759 | -0.149 | 0.882 | 0.882 |
| COL3A1 vs Model | 0.751 | 0.813 | -1.740 | 0.082 | 0.123 |
| MMP1 vs Model | 0.759 | 0.813 | -1.803 | 0.071 | 0.123 |

Table S2. DeLong test for ROC curves of COL3A1, MMP1, and the prediction model in the test set.

| Comparison | AUC1 | AUC2 | Z | P value | Adjust-p value |
| --- | --- | --- | --- | --- | --- |
| COL3A1 vs MMP1 | 0.766 | 0.703 | 0.554 | 0.580 | 0.869 |
| COL3A1 vs Model | 0.766 | 0.766 | 0.000 | 1.000 | 1.000 |
| MMP1 vs Model | 0.703 | 0.766 | -1.048 | 0.295 | 0.869 |

**Supplementary Methods**

**1. Selection of Coagulation-Related Genes via GeneCards Database**
A candidate list of coagulation-related genes (CRGs) was retrieved from the GeneCards database (https://www.genecards.org/, accessed on 2025/10.1). The database’s “Relevance score” is a composite algorithmically-generated metric designed to quantify the association between a gene and a user-defined search term (“coagulation” in this study). It integrates evidence from multiple independent sources, including scientific literature (PubMed), pathway databases (e.g., KEGG, Reactome), protein-protein interaction networks, and gene expression co-occurrence data. A higher score indicates stronger and more diverse evidence supporting the association. We applied a threshold of “Relevance score ≥ 1” to perform an initial screening. This specific threshold was selected to identify genes that are supported by more than a single piece of weak evidence or at least one line of substantial evidence linking them to coagulation processes, thereby constructing a broad yet evidence-based candidate gene set for the subsequent exploratory analysis of coagulation-associated pathology in osteoarthritis (OA).

**2. Data Preprocessing and Differential Expression Analysis**

- Batch Effect Correction: Non-biological technical variation across merged datasets was removed using the removeBatchEffect function from the limma R package (version 3.58.1).
- Microarray Data Normalization: Raw gene expression data from microarray platforms were normalized using the quantile normalization method via the normalizeBetweenArrays function in the limma package.
- Differential Expression Analysis: Differentially expressed genes (DEGs) were identified using the limma package. Genes with an absolute log2 fold change (|log2FC| > 0.7) and an adjusted P-value < 0.05 were considered statistically significant. The P-values were adjusted for multiple testing using the Benjamini-Hochberg False Discovery Rate (FDR) method.

**3. Protein-Protein Interaction (PPI) Network Analysis**

The PPI network for candidate genes was constructed using the STRING database (version 11.5). Interactions with a minimum required interaction (confidence) score of 0.4 were retrieved, which includes both experimentally determined and predicted interactions.

**4. Machine Learning for Feature Selection**

Three machine learning algorithms were implemented using the following R packages and key parameters for feature importance ranking:

- Random Forest (RF): Implemented using the randomForest package (version 4.7-1.1). The model was run with ntree = 500 trees using default settings for other parameters.
- Support Vector Machine (SVM): Implemented using the kernlab package (version 0.9-32) with a Radial Basis Function (RBF) kernel. Hyperparameter tuning (cost parameter C and kernel coefficient gamma) was performed via 5-fold cross-validation using the caret package (version 6.0-94).
- Generalized Linear Model (GLM): Implemented using the glm function (base R) with a binomial family for logistic regression.
  All models were trained and evaluated using the caret framework to ensure consistency.

**5. Gene Set Enrichment Analysis (GSEA)**

Functional enrichment analysis was performed using the clusterProfiler R package (version 4.10.0). The analysis was based on a custom gene set related to coagulation pathways, compiled from public databases. Enrichment significance was assessed through 1000 permutations. A corrected P-value (FDR) < 0.05 was set as the significance threshold.

**6. Statistical Analysis and Reproducibility**

- Multiple Testing Correction: Throughout this study, the Benjamini-Hochberg (FDR) method was applied to control the false discovery rate in all high-throughput analyses involving multiple comparisons (e.g., differential expression, enrichment analysis).
- Software and Version: All analyses were conducted using R software (version 4.3.2). Key package versions are specified above. Detailed analysis scripts are available from the corresponding author upon reasonable request.
